# Primary Ewing’s sarcoma/primitive neuroectodermal tumor of the ileum: case report of a 16-year-old Chinese female and literature review

**DOI:** 10.1186/s13000-017-0626-3

**Published:** 2017-05-04

**Authors:** Teng Li, Fang Zhang, Yarui Cao, Shoubin Ning, Yongmin Bi, Weicheng Xue, Li Ren

**Affiliations:** 1 Department of Pathology, The General Hospital of Air force, PLA, Fucheng Road 30th, Beijing, China; 2 Department of Gastroenterology, The General Hospital of Air force, PLA, Fucheng Road 30th, Beijing, China; 3 Department of Radio and Imaging, The General Hospital of Air force, PLA, Fucheng Road 30th, Beijing, China; 4 Department of Pathology, Beijing Cancer Hospital, Fucheng Road 52nd, Beijing, China

**Keywords:** Ewing’s sarcoma, Primitive neuroectodermal tumor, Extraosseous, Small intestine, FISH, EWS gene

## Abstract

**Background:**

Ewing’s sarcoma (ES) and primitive neuroectodermal tumors (PNET) are closely related tumors. Although soft tissue ES/PNET are common in clinical practice, they are rare in the small intestine. Because of the absence of characteristic clinical symptoms, they are easily misdiagnosed as other benign or malignant diseases.

**Case presentation:**

Here, we present the case of a 16-year-old female who complained of anemia and interval hematochezia. Her serum test results showed only a slight elevation of CA-125 and a low level of hemoglobin. Computer tomography and magnetic resonance imaging revealed a cystic and solid mass in the lower abdominal quadrant and pelvic region, which prompted suspicion of a malignant gastrointestinal stromal tumor of the small intestine. After resection, the tumor’s histology and immunohistochemistry (positive for CD99, vimentin and synaptophysin) results suggested ES/PNET. Fluorescent *in situ* hybridization tests proved the breakpoint rearrangement of the *EWSR1* gene in chr 22.Ultrastructural analysis revealed neurosecretory and glycogen granules in the tumor cell cytoplasm.

**Conclusions:**

Together, these data supported the diagnosis of a rare case of localized ES/PNET in the small intestine without adjuvant chemo- or radiotherapy. To our knowledge, this is the first report from China of a primary small bowel ES/PNET in the English-language literature. In addition, on the basis of findings from previous publications and the current case, the optimal treatment for localized gastrointestinal ES/PNET is discussed.

## Background

Ewing’s sarcoma (ES)/primitive neuroectodermal tumor (PNET) is a small round cell tumor with simple sarcoma-specific genetic alterations resulting in *TET/FET* family member and *ETS* family member fusion proteins [1]. Pathologists no longer categorize ES and PNET as different tumors because their genetic abnormalities overlap. Instead, they are termed the Ewing’s sarcoma family of tumors [2, 3], together with the Askin tumor. ES/PNET are most commonly seen in patients younger than 20 years of age and are derived mainly from bone [4]. The tumor has been discovered in most organs, including the pancreas, liver, adrenal gland, esophagus, and uterus [5–11]. However, ES/PNET is extremely rare in the small bowel. Although it has been reported previously in this location [12–17], none of these reports came from China. Here, we present the first reported case in China of primary ES/PNET in the ileum with EWS rearrangement.

## Case presentation

### Clinical history

**Fig. 1:**
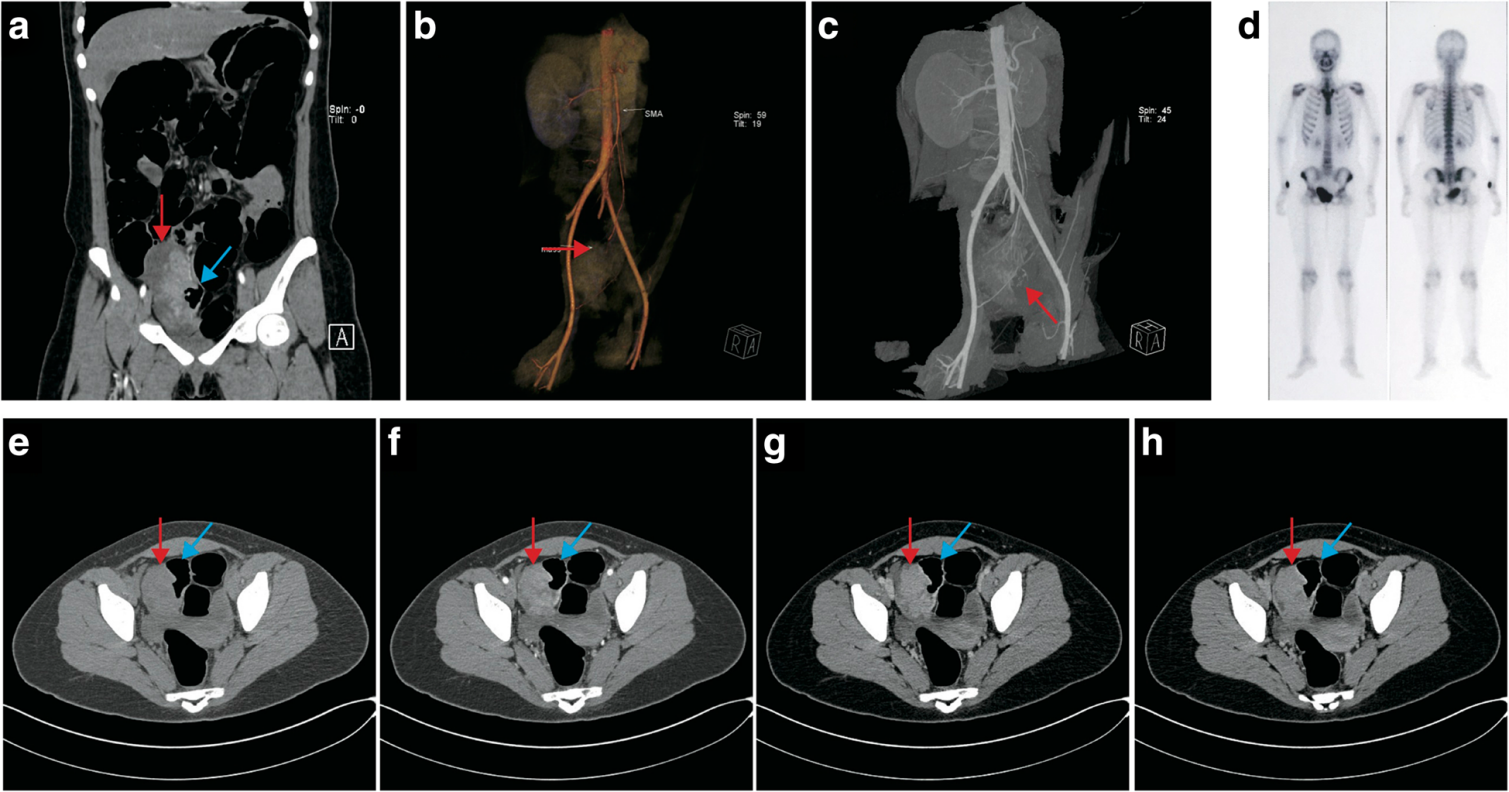
Abdominal and pelvic CT scan, 3D reconstruction and ECT demonstrating the tumor originating in the ileum of the patient. CT scan was performed immediately after enteroscopy. Thus, the patient’s intestine was dilated. **a** Coronal scan arterial phase reveals that the tumor was derived from ileal wall. **b** 3D reconstruction with volume rendering technique illustrates the supporting vasculature. **c** 3D reconstruction with maximum intensity projection demonstrates major vascular support of the tumor. **d** Postoperative bone scintigraphy proved that there was no lesion in her skeletal system. **e** Plain scan revealed pelvic a 10.0 × 7.6 × 5.3 cm mass with areas of necrosis. Most of the mass was had clear boundaries with surrounding tissues, although part of it obliterated the lumen of the terminal ileum. Workup for metastasis was negative. **f**-**h** Contrast-enhanced arterial, venous, and delayed phase pelvic CT scan revealed enhancement of the solid part of the tumor in all phases, with the peak in the arterial phase

A 16-year-old Chinese girl presented complaining of anemia and interval hematochezia. Her hemoglobin was 54 g/L on admission. Capsule endoscopy and double-balloon enteroscopy showed mucosal hyperemia, edema and mass protrusion on the ileal wall. Computed tomography (CT) scans and three-dimensional reconstruction revealed a 10.0 × 7.3 × 5.3 cm irregular mass that had developed from the ileal wall in the right lower quadrant (Fig. 1a-h). The lesion showed intense but inhomogeneous enhancement following contrast administration (Fig. 1e-h), particularly in the arterial phase. There was a small amount of effusion in the pelvic cavity. Pelvic magnetic resonance imaging (MRI) indicated a right ovarian cyst in addition to the above mass. Both CT and MRI prompted suspicion of malignant GIST of the small bowel. Her serum CA-125 was slightly increased (50.5 U/mL, standard 0–36 U/mL), but the other markers were within normal limits. The tumor and a loop of small intestine were resected through a right ventral midline incision. The patient recovered uneventfully. Postoperative bone scintigraphy proved that there was no lesion in the skeletal system (Fig. 1 d). Her chest CT scan and cerebral MRI were also unremarkable. Thus, the patient was classified as T2aN0M0 according to the 8th edition of the AJCC Cancer Staging Manual.

### Gross features

**Fig. 2:**
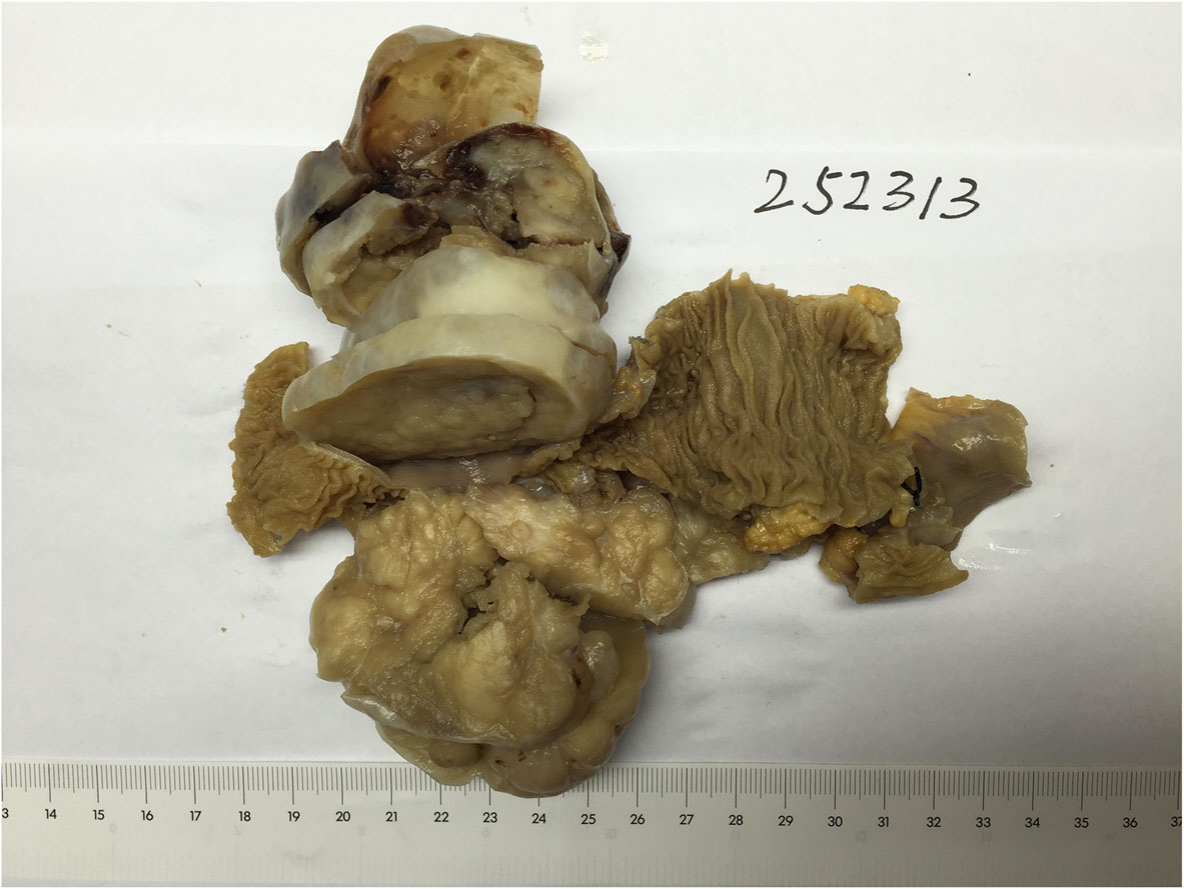
Gross features of the tumor with the resected ileum

On laparotomy, a large cystic and solid mass 10.5 cm in diameter was found arising from the ileal wall. The cut tumor surface showed large central hemorrhagic and necrotic changes and pseudocystic degeneration. The tumor tissue was mostly light gray and solid, with some softer and more friable reddish congested areas (Fig. 2).

### Histological features

**Fig. 3:**
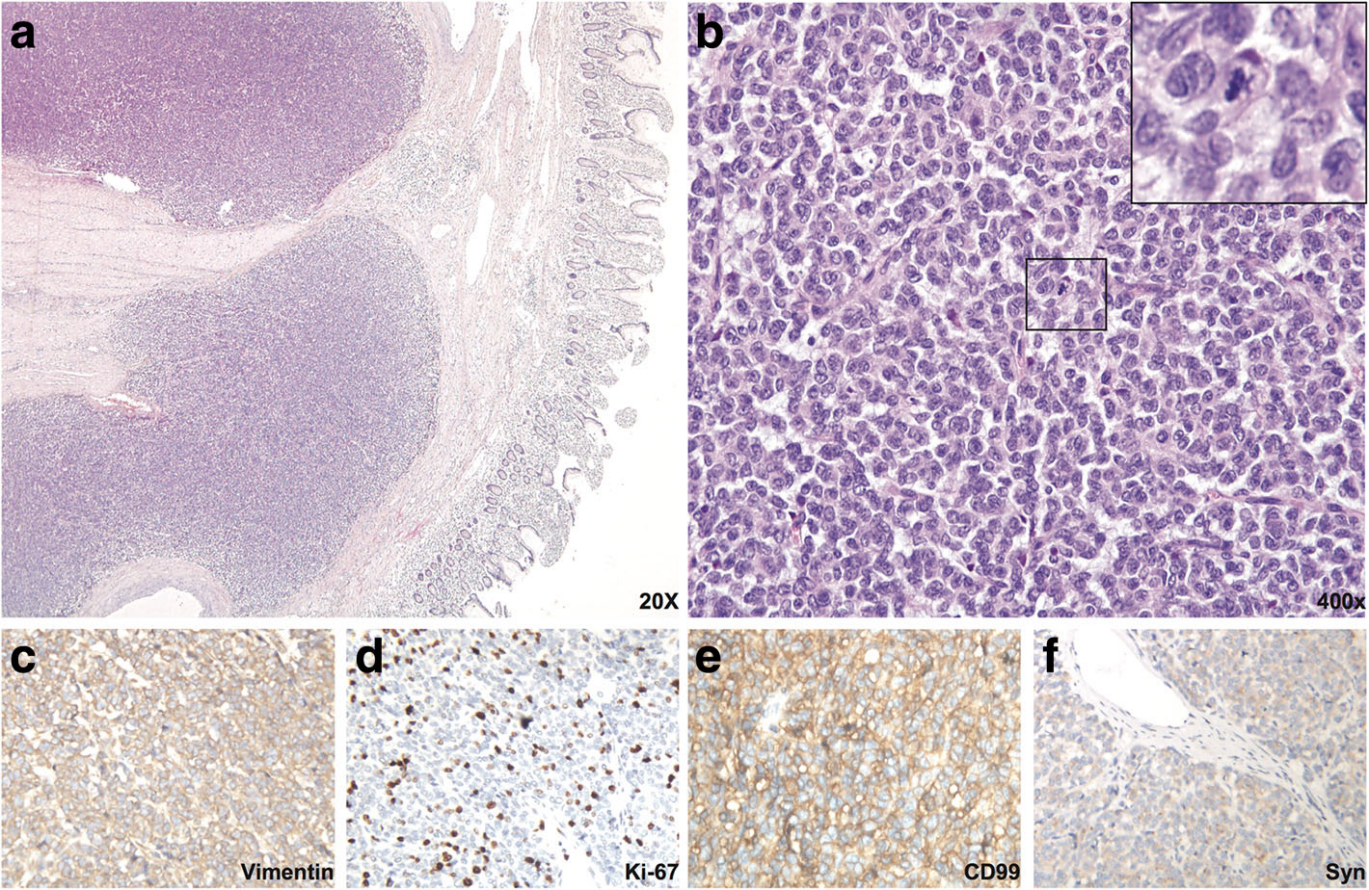
Histological and immunohistochemical features of the intestinal tumor. **a** Low-power view with HE staining indicates sheets of tumor cells invading the myometrium and submucosa. **b** High-power HE view suggests that the tumor cells are small, round and form Homer-Wright structures. The boxed region is amplified in the upper right corner and is used to show pathological mitosis. **c**-**f** The tumor is positively stained for Vimentin, Syn, CD99, and the Ki-67 index is high (~40%). All immunohistochemistry images were taken under 200× magnification

Cross sections revealed solid nests of small round tumor cells arising from the muscular layer and infiltrating all layers of the ileum wall. Cystic and hemorrhagic changes were seen on part of the sections, as were sharply demarcated borders that were frequently covered by intact serosa. No vascular tumor embolus or perineuronal invasion were observed. The serosal layer and the surgical margins of the specimen were free of disease. Under high-power view, tumor cells were round or elliptical, possessing scant eosinophilic cytoplasm and abortive pseudorosette formation. The tumor cell nuclei were round, with exquisite chromatin, ambiguous nucleoli, and 9/10 high-power-field pathological mitoses (Fig. 3a-c). Tumor cells showed positive immunoreactivity for Vimentin and CD99 (Fig. 3 d, j) and moderate staining for Cam5.2, Syn and PR (Fig. 3 f, g, k). Results were negative for CKpan, LCA, S-100, HMB45, Melan-A, CD31, CD34, NSE, P53, CD56, CgA, SMA, Desmin, CD117, Dog-1, ER, Bcl-2, and alpha-inhibin.

### FISH

**Fig. 4:**
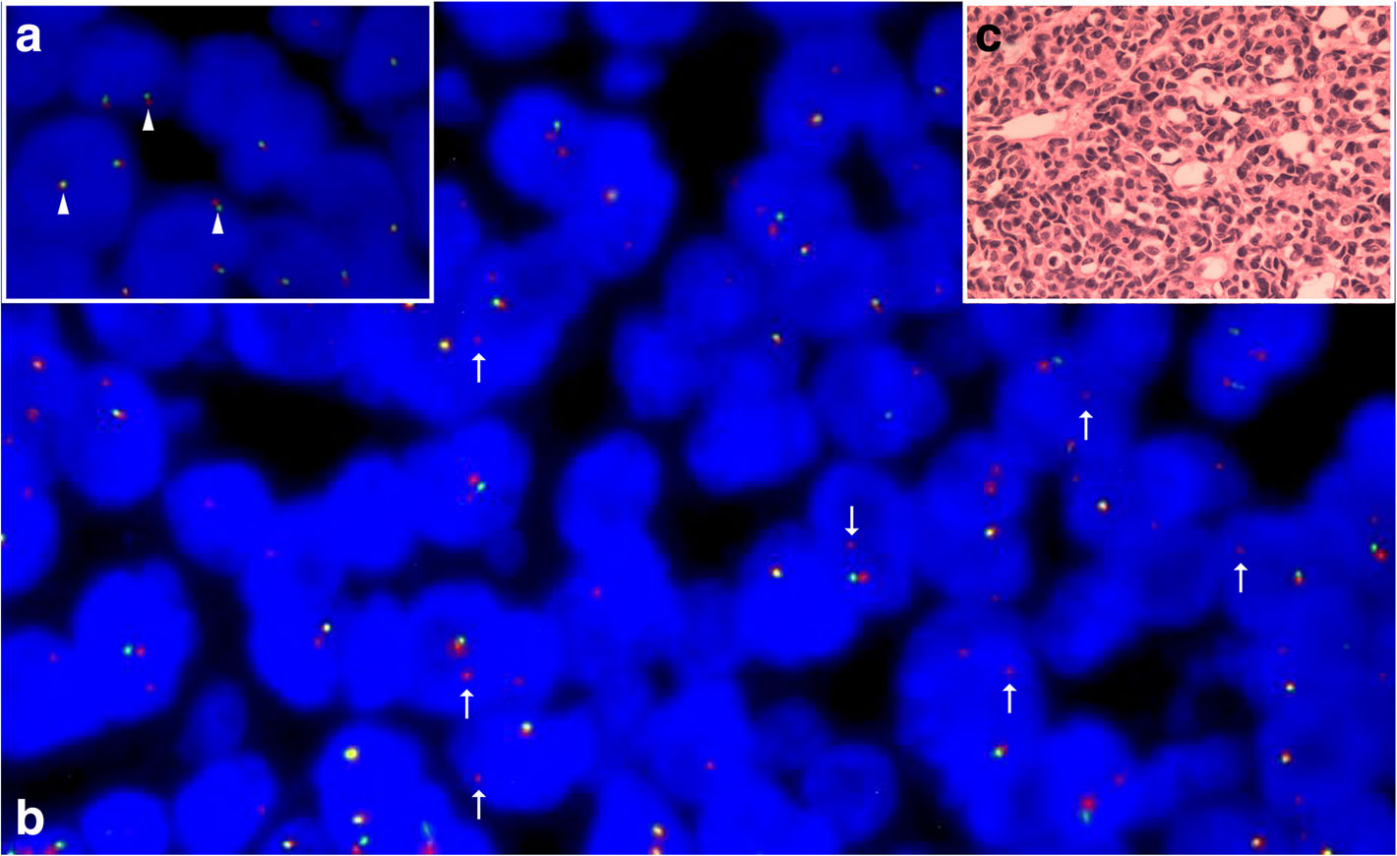
Dual color (*red*/*green*) break-apart probe FISH test of the tumor. **a** Normal karyotype cells have two yellow (*red/green* merged) signals (*arrowheads*). **b** Most tumor cells (90%) had one yellow (*red/green* merged) signal and one red signal (*arrows*). C. Consecutive sections were HE-stained

Dual color break-apart probe FISH examination showed that 90% of the cells (100 counted cells per slide) exhibited 1 yellow and 1 red signal (1F1R) and that 6% of the cells exhibited 1 yellow, 1 red and 1 green (break-apart) signal (1F1G1R). However, only 4% cell had two yellow signals, which proved a break of the *EWSR1* locus (2 F) (Fig. 4a-c).

### EM

**Fig. 5:**
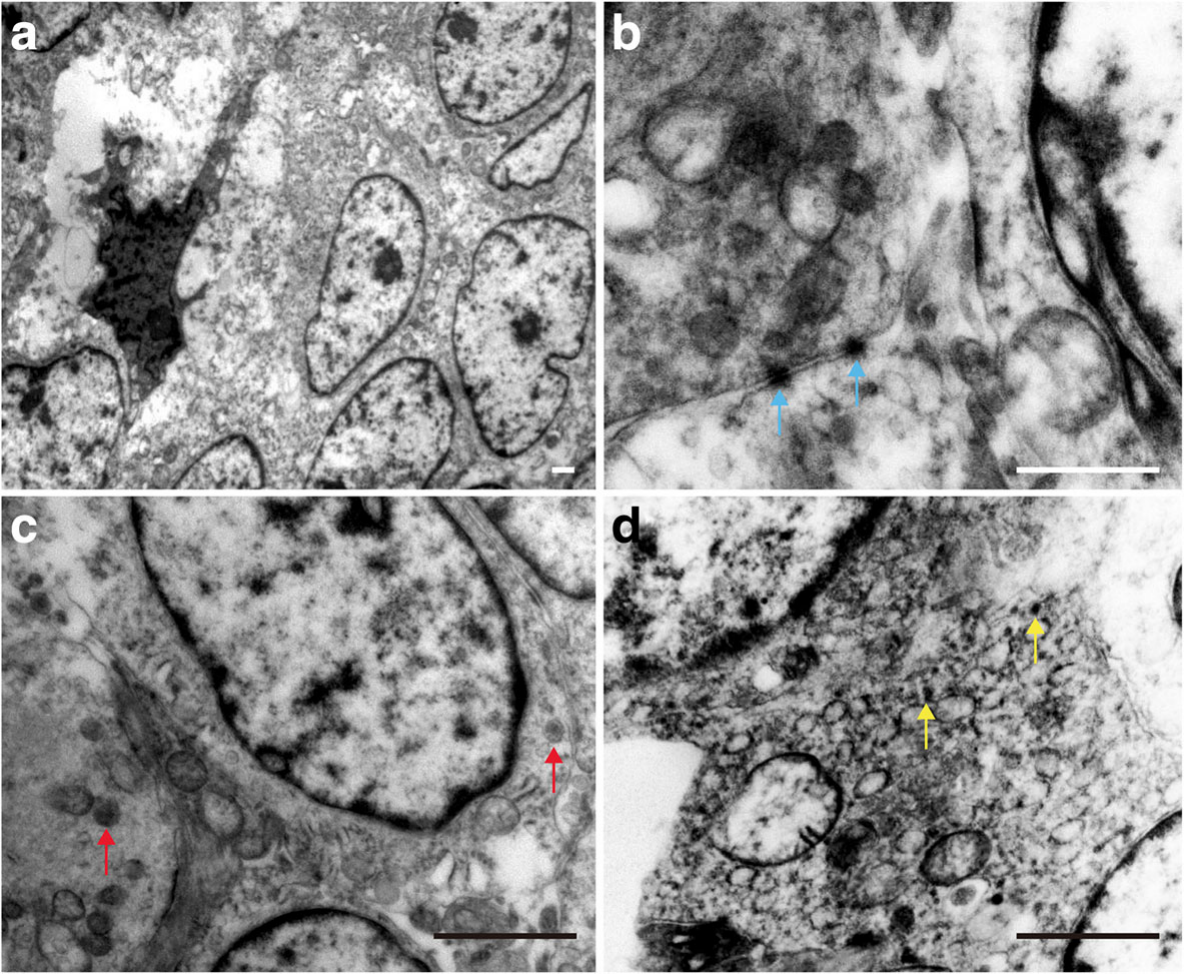
Ultrastructure analysis of the tumor. **a** At lower magnification, EM shows the general tumor ultrastructure. **b** Cell-cell gap junctions (*blue* arrow) were observed in some areas. **c** Neurosecretory granules (*red* arrow) were rarely seen in the tumor cytoplasm. **d** Glycogen granules (*yellow* arrow) existed in most tumor cells. All bars = 2000 nm

Transmission electron microscopy revealed dense clusters of tumor cells, interspersed with a few interstitial cells (Fig. 5 a). The tumor cells were small and irregular, with scant cytoplasm and organelles, and significant nuclear atypia (Fig. 5 a). Some cells had small nucleoli (Fig. 5 a). Occasionally, gap junctions between the cells were observed (Fig. 5 b), but neuroendocrine granules in the cytoplasm were rarely seen (Fig. 5 c). Most cells had glycogen particles attached to the endoplasmic reticulum (Fig. 5 d).

### Treatments and outcome

The patient underwent an exploratory laparotomy, and tumor resection was performed along with 60 cm of ileum. The patient refused chemotherapy and/or radiotherapy as adjuvant treatments. She is currently alive (10 months after the surgery) without any signs of recurrence.

## Discussion

ES/PNET belongs to a family of tumors that harbor the *EWSR1*-*ETS* fusion protein, according to recent studies [18]. It is the second most common pediatric sarcoma of bone. It most commonly arises from bone but can develop in extraskeletal sites [19]. The *EWSR1* gene, together with several other genes, forms the TET family [20]. Their motif of RNA binding activity enables the *EWSR1*-*ETS* fusion protein to regulate target genes as transcription factors [21, 22]. Previous research provided evidence that mesenchymal stem cells may be candidate cells from which ES/PNET originate and that *EWSR1*–*FLI1* may be the sole initiating factor in the pathogenesis of these tumors [20, 23]. Such expression results in cell transformation, with the subsequent emergence of tumors bearing the morphological and gene expression hallmarks of Ewing’s sarcoma [24].

Gastroenterological ES/PNET is extremely rare. Here, we have summarized all previous publications of gastrointestinal ES/PNET in Table 1 [7, 12, 13, 17, 25–49]. Among the 36 cases, 3 cases were derived from the esophagus, 9 from the stomach, 5 were of colorectal origin, and 19 arose from the small intestine. The patient gender ratio (female/male) was 22/14, and the ages ranged from 9 to 68 years. Thirty-one of 32 cases were positive for CD99 immunoreactivity. Fluorescent in situ hybridization or real-time PCR tests confirmed that most cases had the *EWSR1*-*ETS* fusion protein. Intriguingly, however, only 4 non-metastatic gastrointestinal ES/PNET cases were treated only by resection of the tumor. Follow-up of these cases suggested that the patients were relatively younger and had up to 20 months of disease-free survival. In the current case, the young patient also refused to take adjuvant chemo- or radiotherapy. To our delight, after the 10-month follow-up examination, the patient is currently alive and well, without any sign of recurrence.

**Table 1:** Review of reported cases of gastrointestinal ES/PNET

Tumor site	Age	Sex	CgA	Syn	CK	CD99	CD117	FLI1	FISH break-apart EWSR1	RT–PCR EWS–FLI1	Metastasis at diagnosis	Treatments	Follow-up	Reference
Esophagus	44	F	-	-	-	+	-	+	+	+	-	Cx	ND	Johnson AD et al.
Esophagus	56	M	-	ND	-	+	ND	ND	ND	+	Lymph nodes	Sx + ImCx	ND	Maesawa C et al.
Esophagus	21	M	ND	ND	-	-	-	ND	+	ND	-	Sx + Rx	ND	Kim SB et al.
Gastric	31	F	ND	ND	-	+	ND	+	-	+	-	Sx + Rx	3 years DFS	Khuri S et al.
Gastric	19	M	ND	ND	ND	+	+	ND	ND	ND	-	Cx	ND	Aras M et al.
Gastric	41	F	+	+	ND	+	+	ND	ND	+	Intra-peritoneal	Sx + Cx + Rx	Died 110 months after surgery	Inoue M et al.
Gastric	30	M	-	-	-	+	ND	ND	ND	ND	-	Sx	6 month DFS	Ankouz A er al
Gastric	14	M	-	-	-	+	+	ND	ND	+	Liver	Sx + Cx	24 months DFS	Czekalla R et al.
Gastric	55	M	+	+	-	+	+	-	ND	+	Lymph nodes	Sx	13 months DFS	Song JM et al.
Gastric	68	M	-	ND	-	+	+	ND	ND	+	Liver	Sx + Cx	Died 13 months after diagnosis	Rafailidis S et al.
Peri-gastric	44	F	-	-	-	+	+	ND	+	ND	-	Sx	20 months DFS	Colovic RB et al.
Gastric	63	F	ND	ND	ND	ND	ND	ND	+	+	-	Sx + Cx	ND	Maxwell AM er al
Colorectal	59	M	ND	+	-	+	-	ND	ND	+	Peritoneal dissemination	Sx	Died 7 months after diagnosis	Kuwabara K et al.
Colorectal	24	F	-	-	-	+	ND	ND	ND	+	-	Sx	20 months DFS	Tokudome N et al.
Colorectal	17	M	-	-	-	+	ND	ND	ND	+	-	Sx + Cx	1 years DFS	Drut R er al
Colorectal	34	F	ND	ND	-	+	ND	ND	ND	+	Liver	Sx + StemCx	7 years DFS	Aboumarzouk OM et al.
Colorectal	53	M	-	ND	-	+	ND	ND	ND	ND	-	Sx + Cx + Rx	Died 2 years after diagnosis	Vardy J et al.
Small bowel	21	F	ND	-	+	+	ND	ND	ND	ND	-	Sx + Cx	10 months DFS	Adair et al.
Small bowel	20	F	ND	ND	ND	+	ND	ND	+	-	-	Sx + Cx	18 months DFS	Kie et al.
Small bowel	13	M	ND	-	+	+	ND	ND	+	ND	-	Sx	1 years DFS	Sarangarajan etal
Small bowel	40	M	ND	+	-	+	ND	ND	+	ND	Intra-peritoneal	Sx + Cx	Died with recurrence 5 months after diagnosis	Horie and Kato
Small bowel	14	M	ND	-	+	+	ND	ND	+	+	-	Sx + Cx	10 month DFS	Graham et al.
Small bowel	9	F	-	-	+	ND	ND	ND	+	+	-	Sx + Cx	Died 25 months after diagnosis	Shek et al.
Small bowel	53	F	ND	ND	ND	+	ND	ND	ND	ND	-	Sx	ND	Balasubram-anina et al.
Small bowel	63	M	ND	ND	ND	+	+	ND	ND	ND	Adrenal glands + lymph nodes	Sx + Cx	ND	Kim et al.
Small bowel	44	M	ND	ND	-	+	ND	ND	ND	ND	Intra-peritoneal	Sx + Cx	Died 13 months after diagnosis	Sethi and Smith
Small bowel	32	M	ND	ND	ND	+	ND	+	+	ND	-	Sx + Cx	6 months DFS	Rodarte Shade et al.
Small bowel	15	F	ND	ND	ND	ND	ND	ND	+	+	-	Sx + Cx	ND	Vignail et al.
Small bowel	18	M	ND	ND	ND	ND	ND	ND	ND	ND	-	Sx + Cx	ND	Boehm et al.
Small bowel	18	M	+	+	+	+	+	+	+	+	Liver	Sx	Died 8 months after diagnosis	Milione M et al.
Small bowel	20	M	+	+	+	+	+	+	+	+	Liver	Sx + Cx	Died 28 months after diagnosis	Milione M et al.
Small bowel	42	M	+	+	+	+	+	+	+	+	-	Sx + Cx	Died 11 months after diagnosis	Milione M et al.
Small bowel	45	M	+	+	+	+	+	+	+	+	-	Sx + Cx	Died 13 months after diagnosis	Milione M et al.
Small bowel	15	F	+	+	+	+	+	+	+	+	-	Sx + Cx + Rx	28 months DFS	Milione M et al.
Small bowel	57	M	+	+	+	+	+	+	+	+	-	Lost	Lost	Milione M et al.
Small bowel	28	F	+	+	+	+	-	-	+	+	Liver	Sx + Cx	204 months DFS	Milione M et al.

*F* Female, *M* Male, *ND* Not done, *Sx* Surgery, *Cx* Chemotherapy, *ImCx* Immuno chemotherapy, *StemCx* Stem cell based chemotherapy, *Rx* Radiotherapy, *DFS* Disease free survival

To date, the 5-year survival rate of localized ES/PNET is relatively high (65%-75%). However, the outcome for metastatic patients is usually poor (<30%), despite the use of chemo- and/or radiotherapy [50]. Several studies have indicated that localized extraskeletal ES/PNET has a more favorable outcome than skeletal tumors [51, 52]. The optimal management for localized ES/PNET is still debated. The National Comprehensive Cancer Network guidelines recommend that any ES/PNET should be treated with local treatment (surgery and/or radiotherapy) plus chemotherapy [53]. Nevertheless, consistent with our findings in Table 1, others have suggested that complete surgery, if feasible, may be a better option for local disease considering the late side effects of high-dose radiotherapy especially for children [52, 54]. Because small bowel ES/PNET is extremely rare and difficult to cure, our case will contribute to the understanding of the prognosis and determination of optimal management.

In the current case, the 16-year-old female patient was initially misdiagnosed with malignant GIST because of the clinical symptoms and imaging results. To differentiate among ES/PNET, malignant GIST, clear-cell sarcoma, and synovial sarcoma, immunohistochemistry, ultrastructure analysis and FISH tests were performed. Malignant GIST usually expresses CD117, Dog-1 and CD34, which were all negative in this case. Although both synovial sarcoma and ES/PNET could have genetic rearrangements, the regions of these translocations are quite different. In ES/PNET, Chr22 *EWS-FLI* or *EWS-FEV* translocations are commonly reported [16]. However, in synovial sarcoma, *SYT-SSX* translocation is frequently observed [55]. Clear-cell sarcoma could be ruled out by negative immunohistochemistry for HMB45, S-100 and Melan A. A previous study also indicated the necessity of distinguishing from an intraabdominal desmoplastic small round cell tumor (IDSRCT) by histological and immunohistochemical characteristics when ES/PNET occurs in the abdominal cavity [13].

Previous demographic research has suggested that Ewing’s sarcoma is far less frequent in China than in the United States Caucasian population [56]. However, whether this finding is related to genetic background differences remains to be studied. Two recent publications noted a difference in Ewing’s sarcoma occurrence between Caucasian and Hispanic populations [57, 58]. However, they did not include a reason to explain this differences.

## Conclusions

In conclusion, we have described for the first time a rare case of localized ES/PNET occurring in the small intestine in the Chinese population, as confirmed by ultrastructure and genetic analyses. This case, together with previous reports, has expanded the spectrum of tumors in the small intestine.

## Abbreviations

Bcl-2: B cell lymphoma 2
CA-125: Cancer antigen- 125
CD: Cluster of differentiation
CgA: Glycoprotein hormone alpha chain
CKpan: Pan-cytokeratin
CT: Computed tomography
ER: Estrogen hormone receptor
ES: Ewing’s sarcoma
GIST: Gastrointestinal stromal tumor
LCA: Lymphocyte common antigen
MRI: Magnetic resonance imaging
NSE: Neuron-specific enolase
PNET: Primitive neuroectodermal tumor
SMA: Alpha smooth muscle actin
